# PD-L1 Expression and a High Tumor Infiltrate of CD8+ Lymphocytes Predict Outcome in Patients with Oropharyngeal Squamous Cells Carcinoma

**DOI:** 10.3390/ijms21155228

**Published:** 2020-07-23

**Authors:** Nora Wuerdemann, Sibel E. Gültekin, Katharina Pütz, Claus Wittekindt, Christian U. Huebbers, Shachi J. Sharma, Hans Eckel, Anna B. Schubotz, Stefan Gattenlöhner, Reinhard Büttner, Ernst-Jan Speel, Jens P. Klussmann, Steffen Wagner, Alexander Quaas

**Affiliations:** 1 Department of Otorhinolaryngology, Head and Neck Surgery, Klinikstrasse 33, University of Giessen, 35392 Giessen, Germany; Claus.Wittekindt@klinikumdo.de (C.W.); Shachi.Sharma@uk-koeln.de (S.J.S.); b.schubotz@gmail.com (A.B.S.); Steffen.Wagner@hno.med.uni-giessen.de (S.W.); 2 Department of Otorhinolaryngology, Head and Neck Surgery, Medical Faculty, University of Cologne, Kerpener Strasse 62, 50937 Cologne, Germany; christian.huebbers@uk-koeln.de (C.U.H.); hans.eckel@uk-koeln.de (H.E.); jens.klussmann@uk-koeln.de (J.P.K.); 3 Center for Molecular Medicine Cologne (CMMC), University of Cologne, Faculty of Medicine and University Hospital Cologne, Robert-Koch-Str. 21, 50931 Cologne, Germany; 4 Department of Oral Pathology, Faculty of Dentistry, Biskek Caddesi, Emek, University of Gazi, Ankara 06510, Turkey; sibelg@gazi.edu.tr; 5 Institute of Pathology, Kerpener Strasse 62, University of Cologne, 50937 Cologne, Germany; katharina.puetz@uk-koeln.de (K.P.); reinhard.buettner@uk-koeln.de (R.B.); alexander.quaas@uk-koeln.de (A.Q.); 6 Jean-Uhrmacher-Institute for Otorhinolaryngological Research, University of Cologne, Geibelstrasse 29-31, 50931 Cologne, Germany; 7 Institute of Pathology, Langhansstrasse 10, University of Giessen, 35392 Giessen, Germany; stefan.gattenloehner@patho.med.uni-giessen.de; 8 Department of Pathology, GROW-School for Oncology and Developmental Biology, Maastricht University Medical Center, P. Debyelaan 25, 6229 HX Maastrichtthe, The Netherlands; ernstjan.speel@mumc.nl

**Keywords:** oropharyngeal squamous cell carcinoma, human papillomavirus, programmed cell death 1 ligand 1, CD8-positive T-lymphocytes, MHC I, macrophages, tumor micro-environment, prognosis

## Abstract

Carcinogenesis of human papillomavirus (HPV)-related (+) oropharyngeal squamous cell carcinoma (OPSCC) differs from HPV-negative (–) OPSCC. HPV-related immune-escape-mechanism could be responsible for the development and progression of HPV+ tumors and an immunophenotype different from HPV– OPSCC is expected. The purpose of this study was to analyze the expression of programmed cell death protein 1 ligand 1 (PD-L1) and its prognostic relevance in relation to CD8+ tumor infiltrating lymphocytes (TILs) and the major histocompatibility complex (MHC) I expression in OPSCC. We quantified PD-L1 expression on tumor cells (TC) and macrophages and MHC I expression in association to CD8^+^ TILs by immunohistochemistry on tissue microarray derived from 171 HPV+/-OPSCC. HPV-status was determined by p16^INK4a^ immunohistochemistry/HPV-DNA detection. Presence of CD8^+^ TILs, PD-L1 expression on TC, and a more frequent loss of MHC I in HPV+ compared to HPV- OPSCC was detected. A high amount of CD8^+^ TILs in the whole cohort and in HPV+ OPSCC and PD-L1 expression on TC in HPV- OPSCC was associated with favorable overall survival. There was a trend for an improved outcome according to PD-L1 expression (macrophages) in HPV+ OPSCC without reaching statistical significance. CD8^+^ TILs and PD-L1-expression have prognostic impact in OPSCC and might present useful biomarkers for predicting clinical outcome and personalized therapy concepts.

## 1. Introduction

Human papillomavirus (HPV) is accepted as a pivotal risk factor for development of oropharyngeal squamous cell carcinoma (OPSCC) and rising incidences have been reported from several countries [1,2,3]. With a prevalence of more than 80%, high risk HPV16 is the most common type detected in OPSCC [4,5]. Patients with HPV-related OPSCC are characterized by superior locoregional control and favorable outcome in comparison to patients with HPV-negative OPSCC [6]. Further, OPSCC are known to present levels of heterogeneity and immune escape mechanism highly depend on the tumor and its characteristics itself [7,8]. In viral infection, evasion strategies are often employed to escape the host immune system. HPV-related OPSCC are preferentially located in lymphoid tissue (tonsil, base of tongue) and dysregulation of the immune system in their surrounding might play an important role in carcinogenesis.

Programmed cell death protein 1 (PD-1) is an inhibitory molecule expressed by tumor infiltrating T-cells which is capable of suppressing T-cell activation in the presence of its ligand programmed cell death 1 ligand 1 (PD-L1). PD-L1 (also known as B7 homolog 1 (B7-H1)) belongs to the B7 family of co-stimulatory and inhibitory checkpoint molecules [9]. In non-malignant tissue, PD-L1 is expressed on lymphocytes, endothelial cells, and antigen presenting cells (e.g., macrophages). Their binding to the PD-1 receptor on T-cells causes anergy and inhibits T-cell growth and cytokine secretion [10,11]. PD-1 pathway activation is suggested to regulate T-cell responses, maintaining self-tolerance and preventing the development of spontaneous autoimmune diseases [12]. Upregulation of PD-L1 in the tumor environment allows cancer cells to evade the immune system and to avoid apoptosis or lysis induced by effector T-cells (e.g., reviewed in [13]). Expression of PD-L1 has been reported in several carcinomas including non-small cell lung cancer, melanoma, and head and neck squamous cell carcinoma (HNSCC), however with a diverse effect on survival [14,15,16].

In 2016 treatment with the humanized anti-PD-1 antibody (e.g., Pembrolizumab and Nivolumab) for recurrent and metastatic HNSCC was approved by the FDA. The expression of PD-L1 on tumor and immune cells might serve as a biomarker for the therapeutic response to anti-PD-1 antibodies. However, clinical studies have shown either improved overall survival (OS) independent of HPV and PD-L1 expression status or complete lack of response to immunotherapy when targeting PD-1/PD-L1 axis [17,18]. This indicates the existence of other potent immune evasion mechanisms. A high number of tumor infiltrating lymphocytes (CD8+ TILs) has been identified in various malignancies including HNSCC, to be a powerful prognosticator [19,20,21,22]. Downregulation of cell surface expression of the major histocompatibility complex class I (MHC I) is another mechanism allowing cancer cells to evade the immune system. Physiologically, MHC I presents intracellularly produced endogenous or exogenous peptides to CD8+ T-cells. Thereby, it is attributed a decisive role in the immunological screening for malignancies (reviewed in [23]). A total loss of MHC I expression on tumor cells (TC) is described in up to 40% of HNSCC and immune evasion might play a pivotal role in choice of treatment for OPSCC [24]. However, to this point the expression of MHC I in association with CD8+ TILs and PD-L1 expression on TC and macrophages is not well described in HPV-related OPSCC. Therefore, it is important to investigate these tumors in comparison to HPV-negative tumors as immune evasion might play a pivotal role in choice of treatment for OPSCC.

The aim of this study was to evaluate PD-L1 expression on TC and macrophages and MHC I expression on TC in correlation to CD8+ TILs in a well-characterized, retrospective OPSCC patient cohort according to HPV-status and outcome.

## 2. Results

### 2.1. Patient and Tumor Characteristics

Clinicopathological features of patients and their tumors are displayed in Table 1. Median age of the whole cohort was 59 years, whereas in HPV-negative it was 60 and in HPV-related OPSCC patients it was 57 years. For analysis, the study cohort was evaluated as whole and additionally divided into two groups according to HPV status. A total of 32 (19%) patients were diagnosed with an HPV-related (positive for high-risk HPV-DNA and p16^INK4a^ expression) and 138 (81%) with an HPV-negative OPSCC (Table 1). Patients with an HPV-related OPSCC were less frequently smokers and drinkers (each *p* < 0.001) and tumors were predominantly located in the tonsil region (*p* = 0.014).

### 2.2. Distribution of PD-L1, MHC I, and CD8 Expression

**Figure 1:**
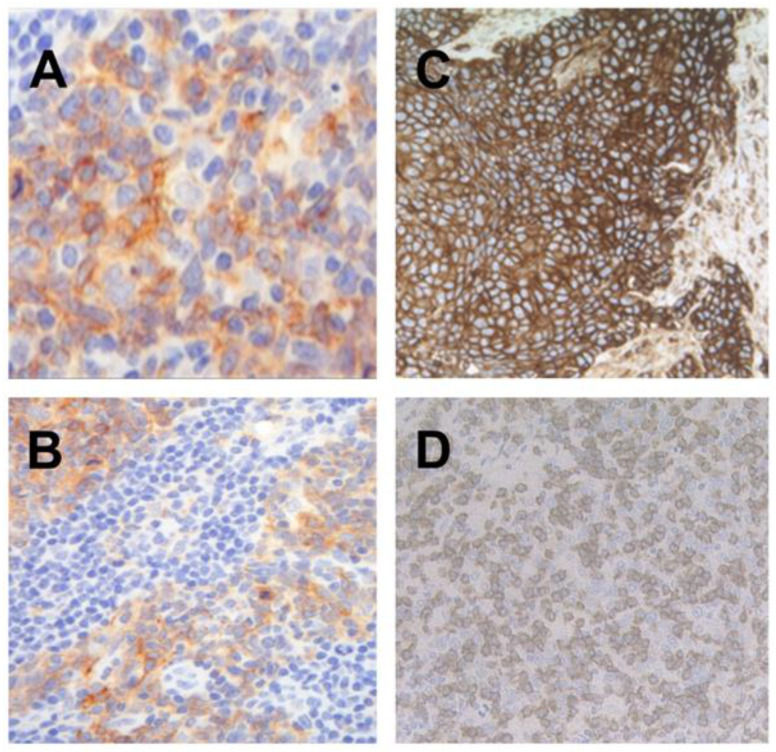
Expression of programmed cell death protein 1 ligand 1 (PD-L1) on tumor cells (TC; (**A**), magnification 200×) and intra- and peritumoral macrophages (**B**), magnification 100×). Strong homogenous staining for major histocompatibility complex class I (MHC I) (**C**), magnification 100×) and membrane-pattern CD8 positive lymphocytes in oropharyngeal squamous cell carcinoma (OPSCC; (**D**), magnification 100×).

Representative images of PD-L1 on TC and immune cells (IC), MHC I and CD8 staining patterns are shown in Figure 1A–D.

Seventy-three percent (73%) of HPV-related tumors presented positive staining for PD-L expression in ≥1% of TC and 27% on macrophages, while it was shown in 33% and 16% of HPV-negative tumors, respectively.

There was a significant association between positive HPV-Status and PD-L1 protein expression on TC (*p* < 0.001, Table 2) but no association between PD-L1 expression on macrophages and HPV-status (*p* = 0.191; Table 2).

Seventy-one percent (71%) of HPV-negative OPSCC presented with MHC I positive staining in ≥20% on TC, but only 50% of HPV-related OPSCC did (*p* = 0.028, Table 2).

A high number of CD8+ TILs was significantly related to HPV-association (*p* < 0.001, 61% vs. 19%; Table 2). No association between PD-L1, MHC I, and CD8+ TILs and other clinicopathological parameters were observed.

There was no significant association for PD-L1 protein expression on TC and macrophages, neither in the whole cohort nor in respect to HPV-status. However, PD-L1 expression on TC was associated with an inflamed tumor microenvironment (CD8+ TILs) in the whole cohort (*p* < 0.001) and according to HPV-status (HPV-related: *p* = 0.028; HPV-negative: *p* = 0.005, Table 2). Further, there was a significant association between PD-L1 expression on macrophages and CD8+ TILs in the whole cohort (*p* = 0.005, Table 2). In HPV-negative OPSCC, PD-L1 expression on TC was significantly associated with MHC I expression on TC (*p* = 0.041, Table 2), but this was not the case in HPV-related OPSCC and the whole cohort, as well.

### 2.3. Survival Analysis and Prognostic Significance

**Figure 2:**
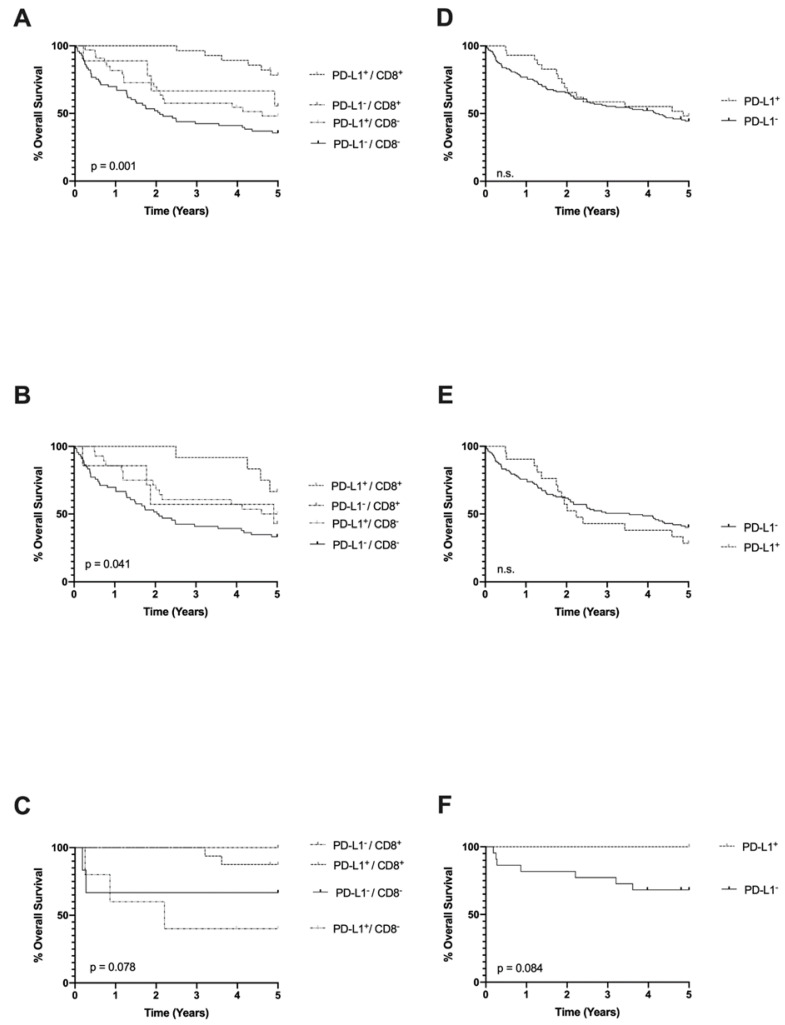
Overall survival according to PD-L1 expression on TC and CD8+ infiltrating lymphocytes in OPSCC in the whole cohort (**A**) in human papillomavirus (HPV)-negative OPSCC (**B**) and in HPV-related OPSCC (**C**). Survival according to PD-L1 expression on macrophages in the whole cohort (**D**), HPV-negative (**E**), and HPV-related (**F**) OPSCC (p-values calculated by log-rank test).

HPV-related OPSCC had a significantly improved overall survival (OS) compared to patients with HPV-negative OPSCC (HR 0.270, CI: 0.125–0.585; *p* = 0.001, Table 3). Further, younger age, low ECOG, and low UICC 7 stage were factors for an improved OS in the whole cohort (HR 1.654, CI: 1.099–2.487; *p* = 0.016; HR 3.386, CI: 2.233–5.134; *p* < 0.001; HR 0.572, CI: 0.364–0.897; *p* = 0.015, Table 3) in univariate analysis. In both OPSCC subcohorts, PD-L1 expression on TC and a high number of CD8+ TILs were factors for an improved OS in univariate analysis, respectively (HR 0.409, CI: 0.255–0.657; *p* < 0.001; HR 0.295, CI: 0.156–0.559; *p* = 0.001, Table 3, Figure 2A). PD-L1 expression on macrophages and MHC I expression on TC showed no impact on OS (Table 3, Figure 2D). Multivariate analysis identified low ECOG and a high number of CD8+ TILs to be the only independent factors for an improved OS in the whole cohort (HR 0.231 CI: 0.144–0.372; *p* < 0.001; HR 3.539 CI: 1.803–6.945; *p* < 0.001, Table 3).

In the HPV-related OPSCC subgroup a high number of CD8+ TILs was the only predictor for an improved OS in uni- and multivariate analysis (HR 5.298 CI: 1.022–27.46; *p* < 0.047, HR 15.099 CI: 2.231–102.2, *p* = 0.005; Table 3, Figure 2C).

In patients with HPV-related OPSCC, PD-L1 expression on TC showed no significant difference in OS (Figure 2C), whereas on macrophages there was a trend towards an improved OS (Figure 2F).

For the HPV-negative OPSCC subgroup, PD-L1 expression on TC, a high number of CD8+ TILs, ECOG and low UICC7 stage remained as independent predictors for an improved OS in univariate survival analysis (HR 2.139 CI: 1.247–3.593; *p* = 0.003; HR 2.268 CI: 1.135–4.603; *p* = 0.021, Figure 2B; HR 0.245 CI: 0.157–0.380; *p* < 0.001, HR 0.583 CI: 0.336–0.927; *p* = 0.023, Table 3). PD-L1 expression on macrophages had no significant impact on OS (Figure 2E). Multivariate analysis identified PD-L1 expression on TC and a low ECOG as independent predictors for an improved OS (HR 1.803 CI: 1.024–3.175; *p* = 0.041, HR 0.231 CI: 0.137–0.338; *p* < 0.001, Table 3).

## 3. Discussion

In this study we demonstrate upregulation of PD-L1 on TC, a high infiltrate of CD8+ TILs and downregulation of MHC I in HPV-related OPSCC. In HPV-related OPSCC a high number of CD8+ TILs was associated with favorable OS in multivariate analysis, whereas in HPV-negative OPSCC high PD-L1 expression on TC was an independent predictor for an improved outcome.

The importance of the human PD-1/PD-L1-axis was first described in the interaction of placental endothelium with fetal proteins to block the maternal inflammatory reaction. The intense interaction of T-lymphocytes, tonsil-crypt epithelia, and foreign, environmental proteins might be comparable to oncogenetic processes. IFN-γ has been demonstrated to upregulate expression of PD-L1 and is also associated with progression of ovarian cancer [25]. Furthermore, an additive effect on transcriptional activation of PD-L1 was shown in mice treated with a combined gene transfer of IFN-γ and p19^Arf^ (syngeneic with human p14^Arf^) [26]. p14^Arf^ is transcribed as an alternate reading frame of CDKN2A, which also encodes p16^INK4a^. In cervical cancers which are virtually exclusively HPV-related, a positive association with HPV type 16 E7 protein and PD-L1 expression was observed [27]. In the oropharyngeal tonsil-crypt cells protect themselves against aggressive T-lymphocytes using the physiological PD-1/PD-L1 interaction. As the palatine tonsils represent the predominant site for HPV-related OPSCC, this mechanism could explain PD-L1 upregulation in our HPV-related, p16^INK4a^-positive OPSCC cohort.

A few studies have been published regarding PD-L1 expression on TC in OPSCC and those also reported higher percentage of PD-L1 on TC in HPV-related OPSCC/HNSCC [14,28,29,30,31,32]. De Meulenaere demonstrated improved OS in the case of PD-L1 expression on TC and a high infiltration of CD8+ cells in the whole cohort of OPSCC [28]. In a study by Tsakiroglou, PD-L1 and CD8+ were investigated in a cohort of 72 OPSCC and proximal expression was related to poor survival only in HPV-negative OPSCC [33]. In our cohort, survival depended predominantly on CD8+ expression independent of HPV-status. Further studies reported no differences in PD-L1 expression on TC according to HPV-status, risk factors, patient/tumor characteristics, or according to OS [34,35].

In mixed HNSCC cohorts with predominantly negative or untested HPV status, PD-L1 expression on TC was associated with poor outcome, whereas in our OPSCC cohort, PD-L1 expression on TC in HPV-negative OPSCC was a predictor for improvedOS [36,37,38,39,40]. PD-L1 expression in oral squamous cell carcinoma (OSCC) investigated in a meta-analysis including 1060 patients, revealed that there was a high variation in results and that PD-L1 expression was not associated with poor survival [41]. A lower number of patients and heterogeneity of HNSCC cohorts, insufficient detection of HPV-status, different cut off values for evaluation, and different therapy regimes in comparison to our cohort, therefore, might explain discrepancy of prognostic value of PD-L1 expression in OPSCC/HNSCC.

Two additional studies investigated PD-L1 expression on immune cells in OPSCC cohorts according to HPV-status [32,42]. They reported a favorable OS in patients with HPV-related OPSCC and PD-L1 expression onimmune cells. This might support our findings indicating a trend for a favorable outcome according to PD-L1 expression on macrophages in the subgroup of HPV-related OPSCC. Still, further research including larger cohorts of HPV-related OPSCC patients is necessary to validate these results.

PD-L1 represents a gatekeeper that regulates the dynamic interaction between CD8+ TILs and TC. The immunosuppressive function of PD-L1 on TC and macrophages can be compensated by the infiltration of CD8+ TILs. In multiple studies including OPSCC the favorable prognostic effect of a high number of CD8+ TILs has been demonstrated [43,44]. We were able to confirm this result in our study demonstrating a positive effect on OS by high infiltration of CD8+ TILs in multivariate analysis in the whole cohort and for HPV-related OPSCC. This is interesting since a high infiltrate of CD8+ TILs is already expected in HPV-related OPSCC due to their predominant location in the palatine tonsil and the base of the tongue. Nevertheless, the higher number of CD8+ TILs still seems to play a pivotal role in a subgroup of HPV-related OPSCC.

Another cell surface molecule that interacts with CD8+ TILs is MHC I, which has been reported to be downregulated in many tumor entities [45,46,47]. MHC I may help TC to escape the host immune system and could be downregulated especially in virus infected cells to avoid elimination by the host immune system. For HPV-related OPSCC it was shown that HPV16-E6 downregulates the expression of MHC I by lymphotoxine and its receptor [48]. Further, E5 is known to prevent transport of HLA-I to the cell surface by interacting with the heavy chain of HLA and therefore retains the complex to the Golgi apparatus and the endoplasmic reticulum [49,50,51,52]. Downregulation of HLA is associated with resistance to CD8+ T cells [51]. In our study we demonstrated MHC I downregulation in a substantial percentage more frequently observed in HPV-related compared to HPV-negative OPSCC. Those results are in line with another study which investigated the role of HLA I subtypes in tonsillar squamous cell carcinoma. Nevertheless, a direct comparison is only possible to a limited extent since this study differentiated between cytoplasmic and membranous staining and investigated subclasses of HLA [53]. Furthermore, this study reported a favorable survival in case of HLA downregulation in HPV-related tonsillar cancer, whereas in our study we were not able to detect an effect on outcome which, however, might be due to the smaller number of patients in this subgroup and different cut-offs chosen. Yoo et al. investigated MHC I expression in association with PD-L1 in a mixed cohort of HNSCC from different sites and independent of HPV status. In the PD-L1 positive group MHC I loss was accompanied by a significant worse survival compared to MHC I positivity. When looking at changes in MHC I expression alone, no effect on OS was seen, which is again in line with our results [53]. As by downregulation of MHC I on TC one would expect worse OS. In a previous study we were able to show that HPV-related OPSCC present with a higher number of cytotoxic natural killer cells (NK-cells) and that a high infiltrate was accompanied by a significantly improved OS [54]. Especially, the downregulation of MHC I seems to be responsible for this effect as it attracts and activates NK-cells which recognize and kill relevant cells [55].

To our knowledge, this is the first study investigating PD-L1 expression on TC and macrophages in association with CD8+ TILs and MHC I expression in a large cohort of OPSCC with precise HPV-status (p16^INK4a^ and HPV-DNA). Although subgroup analyses were sometimes limited due to a smaller number of HPV-related OPSCC cases, we were able to validate existing data in the literature and demonstrate that HPV-related OPSCC are characterized by an immune suppressive tumor microenvironment. Further, we were able to show that especially in HPV-negative OPSCC, PD-L1 expression on TC presents a valuable biomarker. Further studies with extended patient cohorts are necessary to validate these results especially according to MHC I expression and PD-L1 expression on macrophages in HPV-related OPSCC.

Although our study was not aimed to answer whether PD-L1 expression on TC and immune cells can serve as predictive biomarker for immune checkpoint inhibition therapy, we found that PD-L1 protein-expression on TCs is associated with positive HPV-status and is an independent prognostic biomarker especially in HPV-negative OPSCC.

The successful clinical application of PD1/PD-L1 inhibition has proven that (untreated) TC modulate immune checkpoint pathways and can be therapeutically targeted by PD-1/PD-L1 inhibition [10,17,18,56]. Although HPV and PD-L1 present a valuable biomarker to guide treatment decisions in patients with OPSCC, de-escalation trials did not show the desired effect of unchanged survival with less side effects in all patients but might be very successful in specific patient groups. Additional markers are necessary to guide therapy decisions and to determine subgroups, which would benefit from de-escalating therapy regimes. Our data presents new potential biomarkers that might enable further risk stratification of patients with OPSCC. Future studies are necessary to prove the effectiveness of immune checkpoint inhibition therapy in OPSCCs in relation to expression of PD-L1, infiltration of CD8+ TILs, and MHC I expression and possible other markers. Due to discrepant results in existing studies, the predictive power of PD-L1 protein and MHC I expression status as a biomarker needs to be further evaluated in patients with OPSCC with adequate HPV-status incorporated.

## 4. Materials and Methods

### 4.1. Cohort

All patients who were diagnosed with OPSCC (C09, C10, International Classification of Diseases for Oncology (ICD-O)) and treated at the University Hospital Giessen between 2000 and 2009 and who had sufficient pre-therapeutic tissue samples available were included in this study. For the preparation of tissue microarray (TMA) cores, formalin-fixed, paraffin-embedded (FFPE) cancer tissue with a thickness of 2–3 mm was mandatory. Therefore, patients treated with primary chemoradiation were often excluded because diagnostic tumor samples were usually insufficient for the preparation of TMA cores, resulting in 171 suitable samples for analysis. Clinicopathological features of the whole cohort and according to HPV-status are displayed in Table 1. Written, informed consent for medical and scientific purpose was obtained from all patients. The study was conducted in accordance with the Declaration of Helsinki, and the protocol was approved by the Ethics committee of Giessen (AZ 95/15, dated 19 October 2015).

Tumor staging and histological grading was assessed according to the 7th edition of the International Union against Cancer (UICC) TNM classification and the WHO criteria for squamous cell carcinomas of the oral mucosa [57].

### 4.2. HPV-DNA Genotyping and p16^INK4a^ Immunohistochemistry

For retrospective determination of HPV-status, DNA was extracted from variable numbers of FFPE tissue sections depending on the tissue size (10 µm sections, approximately corresponding to 10 × 10 mm tumor tissue) using the DNeasy Blood and Tissue Kit by Qiagen, Hilden, Germany, according to manufacturer’s instructions. Extracted DNA was analyzed for mucosal high-risk HPV-DNA and HPV genotypes (16, 18, 31, 33, 35, 39, 45, 51, 52, 56, 58, 59, 68, 73, and 82) as described previously [58]. p16^INK4a^ expression was detected using the CINtec Histology kit (Roche mtm Laboratories, Mannheim, Germany) according to antibody suppliers’ and standard protocols [59,60,61].

### 4.3. Preparation of Tissue Microarrays

FFPE cancer tissue with a thickness of a least 2–3 mm was mandatory to produce TMA cores. Tissue cylinders with a diameter of 1.2 mm each were punched from tumor tissue blocks using a self-constructed semi-automated precision instrument and embedded in empty recipient paraffin blocks. Then, 4 μm sections were transferred to an adhesive coated slide system (Instrumedics Inc., Hackensack, NJ, USA). Immunohistochemistry (IHC) was performed on freshly cut 4-µm TMA sections.

### 4.4. Immunohistochemistry

Immunohistochemical staining of TMA sections for PD-L1 protein expression was performed using the FDA-approved rabbit monoclonal antihuman PD-L1 antibody, clone 28-8 (Dako/Agilent; Santa Clara, CA, USA; dilution 1:100). Positivity was defined as ≥1% of tumor cells or macrophages with membranous staining of any intensity for PD-L1, respectively.

TMA sections were further stained with the rabbit monoclonal antibody EPR 1394Y (Abcam, UK; dilution 1:200) and mouse monoclonal antibody C8/144B (Dako/Agilent, USA; dilution 1:200) for MHC I and CD8, respectively. Human tonsil tissue served as staining control on each of the TMA slides. All IHC stainings were performed using a Bond Max automated system (Leica Biosystems; Wetzlar, Germany) in accordance with the manufacturer’s protocol. Evaluation of the data was determined semiquantitatively by three pathologists (KP or SEG and AQ). Discrepant results, which occurred only in a small number of samples, were resolved by consensus review.

For PD-L1 expression on TC and macrophages <1% was defined as negative, whereas ≥1% of expression was categorized as positive.

For CD8 expression <50 lymphocytes/mm^2^ were assessed as negative and ≥50 lymphocytes/mm^2^ were defined as positive considering peritumoral and intratumoral distribution.

For MHC I evaluation <20% were assessed as negative, whereas ≥20% was defined as positive.

### 4.5. Statistical Analysis

Statistical analyses were performed using SPSS statistical software (IBM SPSS 25.0, Armork, NY, USA). For evaluation of interdependence of the assessed IHC markers and clinicopathological parameters Fisher’s exact test or Pearson’s Chi-square test were used as appropriate. Overall survival (OS) was calculated from initial date (date of histological diagnosis by routine biopsy) to date of death. Follow-up time of event-free patients was not censored. OS rates were calculated by the Kaplan–Meier method. Significance of differences was calculated by log-rank test. Cox proportional-hazards models were used to estimate hazard ratios (HR) with a confidence interval (CI) of 95% for OS in univariate and multivariate analysis. *p*-values ≤ 0.05 were considered significant for all tests.

## Figures and Tables

**Table 1:** Clinicopathological features of the patient cohort with OPSCC (N = 171).

Risk Factors		All		HPV-Related		HPV-Negative		*p*
		(*n* = 171)	100%	(*n* = 32)	19%	(*n* = 138)	81%	
**Nicotine**	never	27	16%	13	42%	13	9%	**<0.001**
	former/current	142	84%	18	58%	124	91%	
Alcohol	≤ 2 drinks/day	75	56%	27	96%	47	45%	**<0.001 ^a^**
	> 2 drinks/day	58	44%	1	4%	57	55%	
Age	young (< 60 years)	92	54%	18	56%	73	53%	0.732
	old (≥ 60 years)	79	46%	14	44%	65	47%	
Gender	male	139	81%	26	81%	113	82%	0.933
	female	32	19%	6	19%	25	18%	
ECOG	healthy (0-1)	118	69%	23	72%	94	69%	0.719
	sick (2-4)	52	31%	9	28%	43	31%	
**Tumor characteristics**								
Localization	tonsil	90	53%	23	72%	66	48%	**0.014**
	other than tonsil	81	47%	9	28%	72	52%	
Grading	low (G1-2)	66	48%	11	42%	55	50%	0.506
	high (G3-4)	71	52%	15	58%	56	50%	
UICC stages	I - III	64	38%	12	38%	52	38%	0.939
	> III	105	62%	20	62%	84	62%	
T-stage	T1-3	129	76%	26	81%	102	75%	0.455
	T> 3	40	24%	6	19%	34	25%	
N-stage	N0	52	31%	7	22%	45	33%	0.208
	N+	116	69%	25	78%	90	67%	
M-stage	M0	157	95%	29	97%	127	95%	0.664 ^a^
	M+	8	5%	1	3%	7	5%	
Recurrence	no	142	83%	31	97%	111	80%	**0.031 ^a^**
	yes	29	17%	1	3%	27	20%	

*p*-values calculated by Pearson’s chi-square test, asymptotic or ^a^ Fisher´s exact test, each two-sided, significant *p*-values (*p* ≤ 0.05) in bold.

**Table 2:** Relation of PD-L1 (tumor cells and macrophages), MHC I expression and CD8 expression according to each other in the whole cohort and according to HPV-status (*n* = 171).

			PD-L1 Expression (Tumor Cells)				PD-L1 Expression (Macrophages)					MHC I Expression					CD8 Expression				
		yes		no		*p*	yes		no		*p*	yes		no		*p*	yes		no		*p*
**All**		64	41%	93	59%		29	19%	130	81%		99	67%	49	33%		42	73%	113	27%	
**PD-L1 Expression** (**tumor cells**)	yes						14	22%	49	78%	0.370	44	*75%*	15	*25%*	0.064	28	46%	33	54%	**<0.001**
	no						15	16%	76	84%		47	*59%*	32	*41%*		9	11%	73	89%	
**PD-L1 Expression** (**macrophages**)	yes											17	*68%*	8	*32%*	0.898	13	48%	14	52%	**0.005**
	no		*39%*		*61%*							76	*67%*	38	*33%*		26	22%	93	78%	
**MHC I Expression**	yes																25	26%	70	74%	0.718
	no																14	29%	34	71%	
**HPV-association**	yes	22	*73%*	8	*27%*	**<0.001**	8	27%	22	73%	0.191	15	50%	15	50%	**0.028**	19	*61%*	12	*39%*	**<0.001**
	no	42	*33%*	84	*67%*		21	16%	107	84%		84	71%	34	29%		23	*19%*	101	*81%*	
																					
											p^b^					p^b^					p^b^
**HPV-related OPSCC**																					
**PD-L1 Expression** (**tumor cells**)	yes						6	*27%*	16	*73%*	1.000	12	*60%*	8	*40%*	0.209	16	76%	5	24%	**0.028**
	no						2	*25%*	6	*75%*		2	*25%*	6	*75%*		2	25%	6	75%	
**PD-L1 Expression** (**macrophages**)	yes											3	*43%*	4	*57%*	1.000	7	88%	1	12%	0.110
	no											11	*52%*	10	*48%*		11	52%	10	48%	
**MHC I Expression**	yes																12	80%	3	20%	0.060
	no																6	40%	9	60%	
																					
											p					p					p
**HPV-negative OPSCC**																					
**PD-L1 Expression** (**tumor cells**)	yes						8	*20%*	33	*80%*	0.611	8	*20%*	33	*80%*	**0.041**	12	*30%*	28	*70%*	**0.005**
	no						13	*16%*	69	*84%*		13	*16%*	69	*84%*		7	*9%*	67	*91%*	
**PD-L1 Expression** (**macrophages**)	yes											14	*18%*	65	*82%*	0.582^b^	6	*29%*	15	*71%*	0.091
	no											4	*12%*	28	*88%*		13	*14%*	83	*86%*	
**MHC I Expression**	yes																13	*16%*	67	*84%*	0.321
	no																8	*24%*	25	*76%*	

*p*-values calculated by Pearson´s chi-square test, asymptotic or ^b^ Fisher´s exact test, each two-sided, significant *p*-values (*p* ≤ 0.05) in bold.

**Table 3:** Univariate and multivariate survival analysis according to risk factors and tumor characteristics (*n* = 171).

				Univariate				Multivariate			
			N	HR	*CI*		*p* ^a^	HR	*CI*		*p* ^b^
**Risk factors/tumor characteristics**					*Lower*	*Upper*			*Lower*	*Upper*	
PD-L1 Expression (tumor cells)		no	93	1			**<0.001**				n.s.
		yes	64	0.409	0.255	0.657					
PD-L1 Expression (macrophages)		no	130	1			0.532				
		yes	29	0.837	0.480	1.461					
CD8 Expression		no	113	1			**<0.001**	3.539	1.803	6.945	**<0.001**
		yes	42	0.295	0.156	0.559					
MHC I Expression		no	49	1			0.930				
		yes	99	0.979	0.604	1.585					
HPV		HPV-negative	138	1			**0.001**				n.s.
		HPV-related	32	0.270	0.125	0.585					
Age		young (<60 years)	92	1			**0.016**				n.s.
		old (≥60 years)	79	1.654	1.099	2.487					
ECOG		healthy (0–1)	118	1			**<0.001**	0.231	0.144	0.372	**<0.001**
		sick (2–4)	52	3.386	2.233	5.134					
UICC7 stages		I-III	64	1			**0.015**				n.s.
		>III	105	0.572	0.364	0.897					
											
**HPV-related OPSCC**											
PD-L1 Expression (tumor cells)		no	8	1			0.739				n.s.
		yes	22	1.246	0.242	6.340					
PD-L1 Expression (macrophages)		no	22	1			0.306				
		yes	8	35.342	0.038	32725.7					
CD8 Expression		no	12	1			**0.047**	15.099	2.231	102.2	**0.005**
		yes	19	5.298	1.022	27.46					
MHC I Expression		no	15	1			0.553				
		yes	15	1.573	0.352	7.042					
Age		young (<60 years)	18	1			0.117				
		old (≥60 years)	14	3.718	0.720	19.187					
ECOG		healthy (0-1)	23	1			0.321				n.s.
		sick (2-4)	9	0.469	0.105	2.096					
UICC7 stages		I-III	12	1			0.213				n.s.
		>III	20	0.260	0.031	2.162					
											
**HPV-negative OPSCC**											
PD-L1 Expression (tumor cells)		no	84	1			**0.004**	1.803	1.024	3.175	**0.041**
		yes	42	2.139	1.247	3.593					
PD-L1 Expression (macrophages)		no	107	1			0.566				
		yes	21	0.848	0.483	1.489					
CD8 Expression		no	101	1			**0.021**				n.s.
		yes	23	2.268	1.135	4.603					
MHC I Expression		no	34	1			0.673				
		yes	84	1.118	0.666	1.879					
Age		young (<60 years)	73	1			0.052				
		old (≥60 years)	65	1.526	0.996	2.337					
ECOG		healthy (0-1)	94	1			**<0.001**	0.231	0.137	0.338	**<0.001**
		sick (2-4)	43	0.245	0.157	0.380					
UICC7 stages		I-III	52	1			**0.023**				n.s.
		>III	84	0.583	0.336	0.927					

HR hazard ratios estimated by Cox proportional-hazards models; CI 95% confidence interval. ^a^ *p*-values calculated by Log Rank (Mantel-Cox) test; univariate; *p* < 0.05 in bold; N/A: not applicable. ^b^ *p*-values estimated by Cox proportional-hazards models, uni- and multivariate; *p* < 0.05 in bold.
